# Interactions between the Nse3 and Nse4 Components of the SMC5-6 Complex Identify Evolutionarily Conserved Interactions between MAGE and EID Families

**DOI:** 10.1371/journal.pone.0017270

**Published:** 2011-02-25

**Authors:** Jessica J. R. Hudson, Katerina Bednarova, Lucie Kozakova, Chunyan Liao, Marc Guerineau, Rita Colnaghi, Susanne Vidot, Jaromir Marek, Sreenivas R. Bathula, Alan R. Lehmann, Jan Palecek

**Affiliations:** 1 Genome Damage and Stability Centre, University of Sussex, Brighton, United Kingdom; 2 Functional Genomics and Proteomics, Masaryk University, Brno, Czech Republic; 3 Central European Institute of Technology, Masaryk University, Brno, Czech Republic; 4 National Centre for Biomolecular Research, Masaryk University, Brno, Czech Republic; National Cancer Institute, United States of America

## Abstract

**Background:**

The SMC5-6 protein complex is involved in the cellular response to DNA damage. It is composed of 6–8 polypeptides, of which Nse1, Nse3 and Nse4 form a tight sub-complex. MAGEG1, the mammalian ortholog of Nse3, is the founding member of the MAGE (melanoma-associated antigen) protein family and Nse4 is related to the EID (E1A-like inhibitor of differentiation) family of transcriptional repressors.

**Methodology/Principal Findings:**

Using site-directed mutagenesis, protein-protein interaction analyses and molecular modelling, we have identified a conserved hydrophobic surface on the C-terminal domain of Nse3 that interacts with Nse4 and identified residues in its N-terminal domain that are essential for interaction with Nse1. We show that these interactions are conserved in the human orthologs. Furthermore, interaction of MAGEG1, the mammalian ortholog of Nse3, with NSE4b, one of the mammalian orthologs of Nse4, results in transcriptional co-activation of the nuclear receptor, steroidogenic factor 1 (SF1). In an examination of the evolutionary conservation of the Nse3-Nse4 interactions, we find that several MAGE proteins can interact with at least one of the NSE4/EID proteins.

**Conclusions/Significance:**

We have found that, despite the evolutionary diversification of the MAGE family, the characteristic hydrophobic surface shared by all MAGE proteins from yeast to humans mediates its binding to NSE4/EID proteins. Our work provides new insights into the interactions, evolution and functions of the enigmatic MAGE proteins.

## Introduction

The SMC5-6 protein complex is one of the three SMC (Structural maintenance of chromosomes) protein complexes present in all eukaryotes. The core of each complex is a SMC protein heterodimer, which is associated with other non-SMC proteins. In the yeasts SMC5-6 is essential for proliferation as well as being involved in the response to different types of DNA damage [1]. It is required to resolve recombination structures [2]–[4] as well as having an early role in the recombination process in response to replication stalling [5]. In human cells it is required for loading cohesin at sites of double-strand breaks [6] and for telomere maintenance via the alternative lengthening of telomeres (ALT) pathway [7].

In the yeasts SMC5-6 is comprised of 8 components [8]–[10]. We and others have shown that there are three sub-complexes. In the Smc6-Smc5-Nse2 sub-complex, Nse2/Mms21 is a SUMO ligase and associates with Smc5 [9], [11]. The crystal structure of the heterodimer of Nse2 and the interacting fragment of Smc5 has been reported recently [12]. The Nse1-Nse3-Nse4 sub-complex bridges the head domains of the Smc5-Smc6 heterodimer [8], [10], [13]. Nse4 is the kleisin component of the complex, but Nse3 also binds both Smc5 and Smc6 globular head domains [13]. Nse1 is a RING finger protein with ubiquitin ligase activity [14]. The third sub-complex is made up of Nse5 and Nse6 [10], [13], which are less well conserved than the others and there is no obvious sequence identity between them in *Saccharomyces cerevisiae* [15] and *Schizosaccharomyces pombe* [10].

With the exception of Nse5 and Nse6, conserved human orthologs of all the SMC5-6 components have been identified and characterised. Nse3 is related to the MAGE (Melanoma-associated antigen) family of proteins [16], [17]. Members of this large protein family have a conserved MAGE-homology domain (MHD). The family is sub-divided into two types. Genes encoding Type I MAGEs (A, B and C sub-families) are expressed only in testis and cancer cells, whereas type II MAGEs are expressed in most tissues. We showed previously that MAGEG1 is the only MAGE protein present in the human SMC5-6 complex and is therefore the ortholog of Nse3 [18]. MAGEG1 has been shown recently to stimulate the E3 ligase activity of human NSE1 [14]. The function of the other MAGE proteins is relatively poorly understood, though there is evidence that several of them are involved with brain development, apoptosis and differentiation [17].

In this paper, we explore the interaction between Nse3 and Nse4 and we identify a conserved hydrophobic pocket on the modelled structure of yeast Nse3 which mediates the interaction with Nse4. We show that the Nse3-Nse4 interaction is conserved in human cells, and that interaction of NSE4b, one of the mammalian orthologs of Nse4, with MAGEG1 results in transcriptional activation in a reporter system. We expand these findings to show that many of the human MAGE proteins are able to react, not only with NSE4a and 4b, but also with related proteins of the EID family.

## Results

### Interactions of Nse1, Nse3 and Nse4

**Figure 1:**
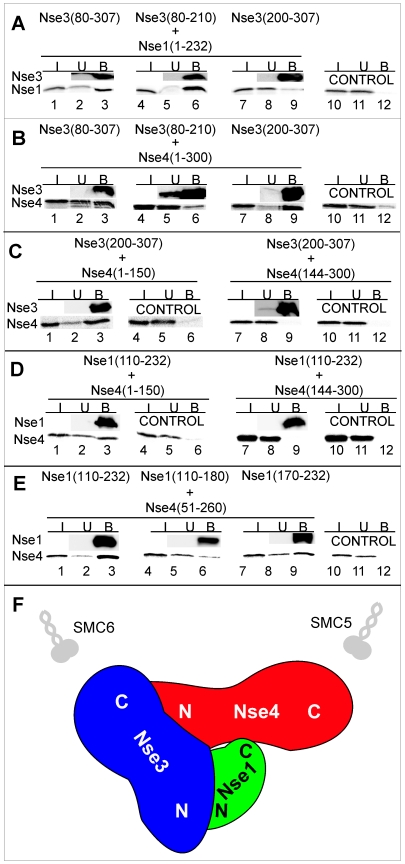
Interactions between Nse1, Nse3 and Nse4. The indicated His-S-tagged fragments of Nse3 (A, B, C) or Nse1 (D, E) were bound to S-protein agarose-beads and then incubated with *in vitro* translated Nse1 (A) or Nse4 (B–E). The reaction mixtures were analysed by SDS–12% PAGE gel electrophoresis. The amount of His-S-tagged protein was analysed by immunoblotting with anti-His antibody and the *in vitro* translated proteins were measured by autoradiography. I, input (5% of total); U, unbound (5%); B, bound (40%). Control, no His-S-tagged protein present. (F) Cartoon of interactions based on panels A–E and our previous work [13].

In our previous studies, we showed that Nse1, Nse3 and Nse4 (originally Rad62) form a sub-complex of the yeast SMC5-6 octameric complex. We wished to gain a deeper understanding of the detailed interactions within this sub-complex. Previously, we showed that the N-terminal half of Nse1 bound to Nse3 [8]. The MHD of the 328 aa protein Nse3 is comprised of aa 90 to 301. Figure 1A shows that, in pull-down assays, S-tagged fragment (aa 80 to 210) containing the N-terminal part of the MHD is sufficient for binding to *in vitro* translated full-length Nse1 (lane 6), whereas the C-terminal 107 aa do not bind (lane 9). Conversely the C-terminal fragment of Nse3 binds to Nse4 whereas the N-terminal part does not (Figure 1B, compare lanes 9 and 6). With Nse4, the N-terminal half of Nse4 is sufficient for binding to the C-terminal part of Nse3 (Figure 1C, lane 3) and the C-terminal part of Nse1 (Figure 1D, lane 3), whereas we showed previously that the C-terminal half of Nse4 binds to Smc5 [13]. Good interaction could also be obtained between Nse4 (aa 51 to 260) and Nse1. Using the latter Nse4 construct, we showed that deletion of the RING finger (located between aa 180 and 232) from Nse1 reduced the interaction with Nse4 (Figure 1E, lane 6), as also found by Pebernard et al [19]. These interactions are shown pictorially in Fig. 1F.

### Interaction of Nse3 and Nse4

**Figure 2:**
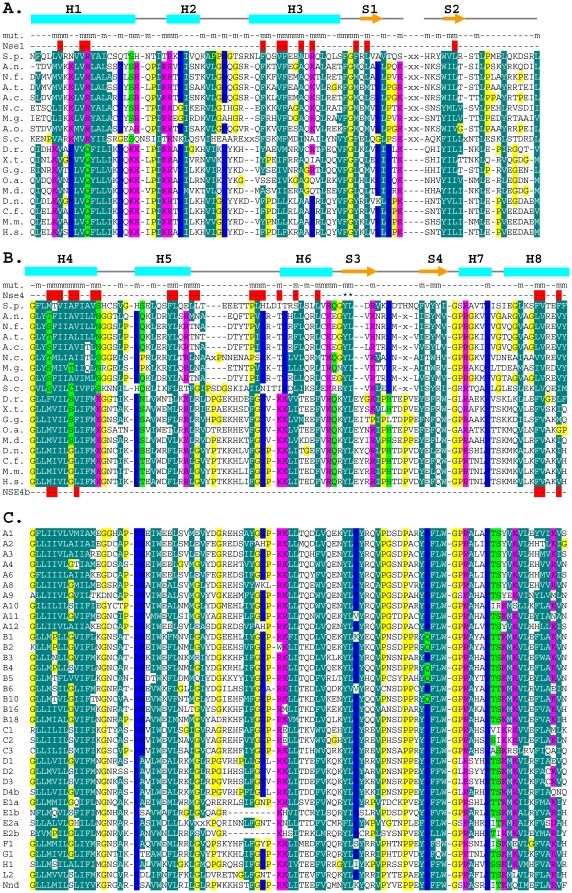
Conserved amino acid residues within the MAGE protein family. Alignment of the N-terminal (A) (aa 89 to 199 of *S.p.* Nse3) and C-terminal (B) (aa 211 to 301 of *S.p.* Nse3) part of MHD domain of Nse3/MAGEG1 subfamily. The Nse3/MAGEG1 orthologs are from *S. pombe* (*S.p.*), *Aspergillus nidulans* (*A.n.*), *Neosartorya fischeri* (*N.f.*), *Aspergillus terreus* (*A.t.*), *Aspergillus clavatus* (*A.c.*), *Neurospora crassa (N.c.)*, *Magnaporthe grisea* (*M.g.*), *Aspergillus oryzae* (*A.o.*), *S. cerevisiae* (*S.c.*), *Danio rerio* (*D.r.*), *Xenopus tropicalis* (*X.t.*), *Galus galus* (*G.g.*), *Ornithorhynchus anatinus* (*O.a.*), *Monodelphis domestica* (*M.d.*), *Dasypus novemcinctus* (*D.n.*), *Canis lupus familiaris* (*C.f.*), *Mus musculus* (*M.m.*), *Homo sapiens* (*H.s.*). Secondary structure derived from the 3D-structure model of Nse3 is indicated above the alignment: cyan rectangle, helix; orange arrow, beta-sheet. Most of the conserved residues were mutated (mut) in the *S. pombe* Nse3 sequence to alanine; m, mutated residue; red rectangles indicate Nse1- and/or Nse4-specific mutants, respectively; Y264 and L265 residues are labelled with asterisk (B). NSE4b-specific residues of MAGEG1 protein are also indicated in red below the MAGEG1 sequence (B). (C) Alignment of C-terminal part of MHD domain of human MAGE proteins. Shading represents amino acid groups conserved across the family: *dark green*, hydrophobic and aromatic; *light green*, polar; *blue*, acidic; *pink*, basic; all glycine and proline residues are highlighted in *yellow*.

**Figure 3:**
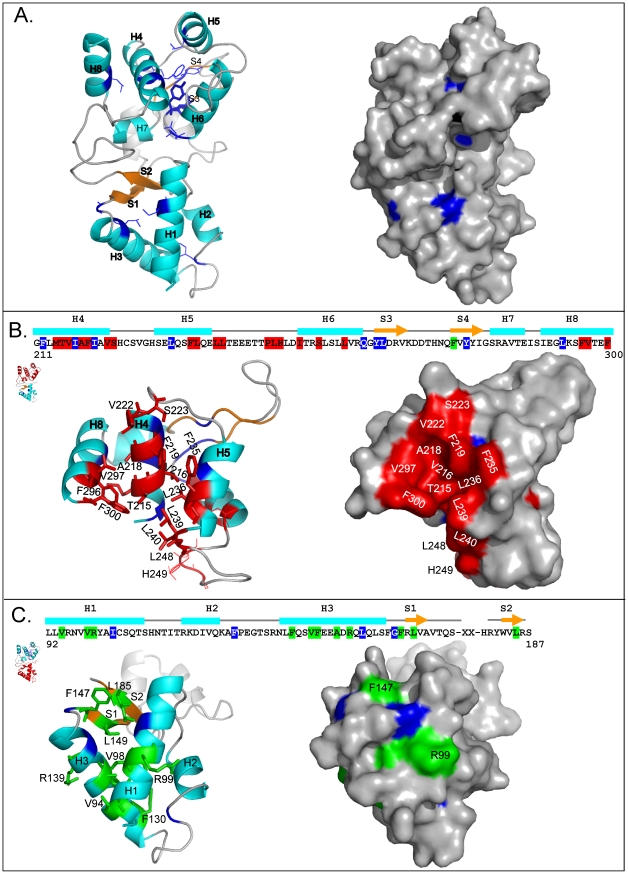
Interacting residues of Nse3 modelled on the crystal structures of MAGEA4 (PDB 2WA0) and MAGEG1 (PDB 3NW0). Homology modelling was used to generate the predicted *S. pombe* Nse3 MHD structure. Ribbon representation (left panels) of the predicted Nse3 3D structure model with helices (cyan) and beta-sheets (orange) indicated as in Figure 2. Right panels represent surface views. (A) The residues that, when mutated, lost their ability to interact with both Nse1 and Nse4 interacting partners are buried inside (indicated in dark blue). (B) Sequence and structure of Nse3 (aa 211 to 300) showing which mutations inhibit interaction with Nse4 (red). Top view of the structure shown in panel (A). (C) Residues in the N-terminal domain (aa 92 to 187) that, when mutated, reduce the interaction with Nse1 are indicated in green. The small cartoons at the left of the panels are miniatures of the full-length structure. The parts indicated in red are expanded in the main panels.

To gain further insight into the interaction surfaces, we have mutated most of the conserved residues in the *S. pombe* Nse3 MHD region (aa 93 to 301, Supplementary Table S1, Figures 2A and B). Each Nse3 mutant was tested for its ability to interact with both Nse1 and Nse4 using the yeast two-hybrid system. 37 out of a total of 82 mutants exhibited no defect in binding to either Nse1 or Nse4 (Table 1). In contrast, 13 mutants lost the ability to interact with both partners. The other 32 mutants disrupted interaction with either Nse1 or Nse4. In order to interpret these data we modelled the structure of Nse3 on the structures of the MHD of MAGEA4 (PDB entry 2WA0) and the recently published structure of MAGEG1 (PDB entry 3NW0 [14]). The structure (Figure 3) is comprised of two domains of approximately equal size, the N-terminal domain being made up of three alpha helices (H1 to H3) and two beta sheets (S1 and S2), whereas the C-terminal domain comprises five helices (H4-8) and two beta sheets (S3 and S4).

**Table 1:** Interaction of *S.pombe* Nse3 mutants with Nse1 and Nse4.

Mutation	Nse1	Nse4	Phenotype	Mutation	Nse1	Nse4	Phenotype
**WT**	+	+	WT	**M214A**	+	−	
**L93A**	+	+		**T215A**	+	+/−	WT
**V94A**	−	+		**V216A**	+	−	
**R95A**	+	+		**I217A**	−	−	WT
**V98A**	−	+		**A218G**	+	−	
**R99A**	−	+	WT	**F219A**	+	−	
**Y100A**	+	+		**I220A**	−	−	
**I102A**	−	+/−		**V222A**	+	−	WT
**Q105A**	+	+		**S223A**	+	−	
**S107A**	+	+	WT	**V227A**	+	+	
**H108A**	+	+		**H229A**	+	+	WT
**N109A**	+	+		**L232A**	−	−	
**T112A**	+	+	WT	**F235A**	+	−	WT
**R113A**	+	+		**L236A**	+	−	WT
**K114A**	+	+		**E238A**	+	+	
**K119A**	+	+		**L239A**	+	−	
**F121A**	−	+/−		**L240A**	+	−	
**E123A**	+	+		**P247A**	+	−	
**T125A**	+	+		**L248A**	+	−	
**R127A**	+	+		**H249A**	+	−	
**F130A**	+/−	+		**I252A**	+	−	
**Q131A**	+	+		**S255A**	+	−	WT
**V133A**	+/−	+		**S257A**	+	+	WT
**F134A**	−	+		**L259A**	+	−	
**E135A**	+	+		**V260A**	+	+	
**E136A**	+	+		**R261A**	+	+	
**A137K**	−	+		**Q262A**	−	−	WT
**R139A**	+/−	+		**Y264A**	−	−	WT
**Q140A**	+	+		**L265A**	−	−	WT
**L141A**	−	+/−	WT	**R267A**	+	+	
**S144A**	+	+	WT	**F276A**	−	+	WT
**F145A**	+	+	WT	**Y278A**	−	−	
**G146A**	−	+/−		**Y279A**	+	+	
**F147A**	−	+		**E287A**	+	+	WT
**L149A**	−	+		**L293A**	−	−	
**V152A**	+	+		**F296A**	+	−	
**S155A**	+	+		**V297A**	+	−	
**H180A**	+	+		**F300A**	+	−	
**Y182A**	+	+		**F301A**	+	+	
**V184A**	+	+		**SUMMARY**	**Structural**		**13**
**L185A**	−	+			**Nse4-specific**		**20**
**T188A**	+	+			**Nse1-specific**		**12**
**L199A**	+	+			**No disruption**		**37**
**F212A**	−	−			**Total**		**82**

Combinations of the indicated mutant Nse3 proteins with Nse1 or Nse4 were analysed in the Y2H system. + and − signify whether or not an interaction was detected. Some of the mutations were introduced into the *S. pombe* genome and sensitivity to MMS and HU was analysed. WT indicates no sensitivity.

Most of the 13 mutations that destroy interactions with both Nse1 and Nse4 change residues that are buried inside the Nse3 molecule (Fig. 3A). These amino acid residues most likely maintain the tertiary structure of the MHD. A group of 20 Nse3 mutants specifically disrupt binding to Nse4 (Table 1), whilst the Nse3-Nse1 interaction remains undisturbed. Consistent with our pull-down results (Fig. 1B) all these mutations cluster in the C-terminal part of Nse3 (Figure 2B). Based on the sites of these mutations, we deduce that the major part of the Nse4-binding site is formed by hydrophobic residues that are well-conserved in helices H4 (M214, T215, V216, A218, F219, V222, S223), H5 (F235, L236) and H8 (F296, V297, F300). Less well conserved residues from the loop region between helices H5 and H6 (L239, L240, L248, H249) may contribute to the binding as well (Fig. 3B). The hydrophobic pattern of these helices is well conserved within the Nse3/MAGEG1 subfamily (Figure 2B) as well as across the whole MAGE family (Figure 2C; see below). We conclude that the interaction with Nse4 is mediated by a conserved hydrophobic pocket on the Nse3 surface (Fig. 3B, right panel).

### Interaction between Nse3 and Nse1

We have identified twelve mutations that specifically destroy the interaction with Nse1 (Table 1). Consistent with the pull-down results (Fig. 1A) most of them cluster within the N-terminal domain of the Nse3 molecule (Figure 2A). Amino acid residues R99, R139 and F147 protrude on the surface, whereas the other residues are partially or fully buried inside the structure of Nse3 (Fig. 3C). We suggest that the latter mutations might disturb the structure of the N-terminal sub-domain of the MHD, while leaving the C-terminal sub-domain containing the Nse4 interaction surface intact. Consistent with our results, human MAGEG1 residues Q94 and F138 (corresponding to R99 and F147 residues in yeast Nse3) contact the NSE1 surface in the 3NW0 co-crystal [14].

**Figure 4:**
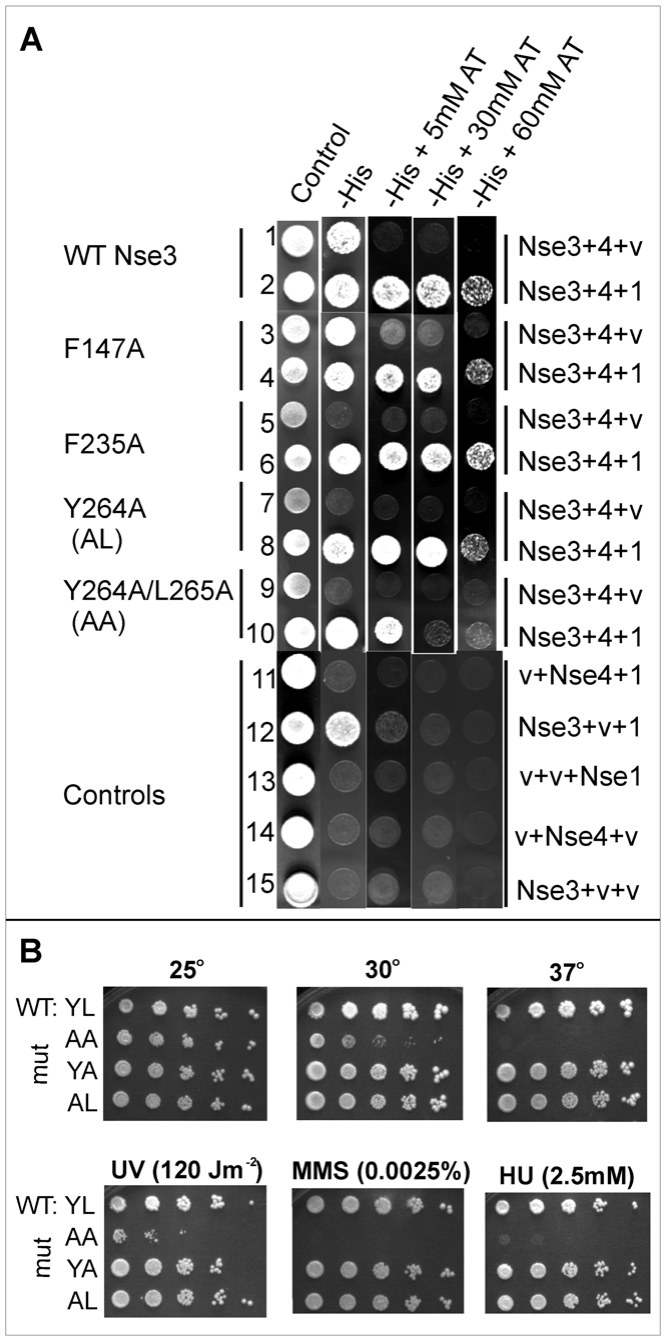
Effect of Nse1 on the interaction between *S.pombe* Nse3 and Nse4. (A) Yeast-2-hybrid plasmids expressing Nse3, either wild-type or mutated as indicated, fused to the Gal4 DNA-binding domain, and wild-type Nse4 fused to the Gal4 activation domain, were co-expressed with either empty vector (v) or Nse1 (1) in yeast cells, which were subsequently plated in the indicated media and grown at 30°C. AT, 3-aminotriazole. (B) Spot tests of Nse3 wild-type cells (wt:YL), Y264A/L265A (AA), L265A (YA) and Y264A (AL) plated under the indicated conditions.

We next analysed the effect of Nse1 on the interaction between mutant Nse3 and Nse4 using a yeast-3-hybrid system. In this system, the interaction between Nse3 and Nse4 allowed growth of the cells in the absence of histidine, but not in the presence of 3AT (Figure 4A, row 1). The addition of Nse1 into the system permitted growth in the absence of histidine and in the presence of up to 60 mM 3AT, indicating a much stronger interaction in the presence of Nse1 (Figure 4A, row 2). We analysed the effects of three single mutations in Nse3 that respectively prevented interaction with Nse1 (F147A), Nse4 (F235A) or both (Y264A) in the Y2H system (Fig. 4A, rows 3, 5 and 7) as well as the double mutant Y264A/L265A (row 9). When Nse1 was also present, the interactions between the single mutants of Nse3 and Nse4 (Rows 4, 6 and 8) were indistinguishable from that between wild-type proteins (Row 2). Furthermore the presence of Nse1 partially restored the interaction between the double mutant and Nse4 (Row 10). We conclude that Nse1 markedly strengthens the Nse3-Nse4 interaction (Fig. 1F).

### Phenotypic analysis of Nse3 mutants

We have integrated 19 of the mutations into the genome of *S. pombe* and analysed the phenotype for temperature-sensitivity as well as for sensitivity to UV light, methyl methanesulfonate (MMS) and HU (Table 1). Because the effects of the mutations on Nse3-Nse4 interaction were largely mitigated by the presence of Nse1 we did not anticipate strong phenotypic effects of the integrated mutations. Indeed, we found that all the single mutants had a normal phenotype, including Y264A and L265A (Figure 4B, rows 3 and 4). Only when we constructed the double mutant Y264A/L265A was there sensitivity to high temperature, UV irradiation, MMS and HU (Figure 4B, row 2). We conclude from this that the interactions in the context of the whole SMC5-6 complex are considerably stronger than those between two components in isolation. In the former context, mutations that disrupt the two-way interactions are insufficient to cause dissociation within the whole complex.

### Interactions between MAGEG1 and NSE4b

**Figure 5:**
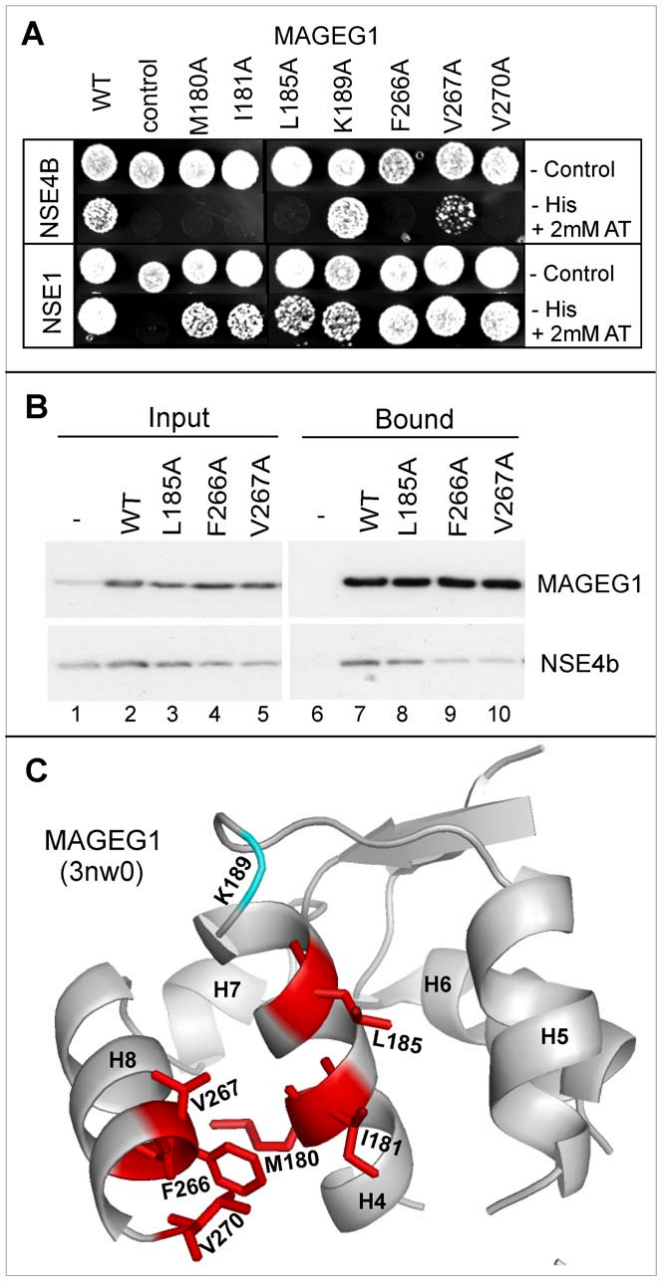
Human MAGEG1 binds NSE4b through conserved hydrophobic surface. (A) Yeast-2-hybrid analysis of the interaction between the indicated mutants of MAGEG1 (aa 55 to 292) and NSE4b (aa 1 to 333) or NSE1 (aa 1 to 266). Interactions result in growth on -Leu,-Trp, -His plates +2 mM AT. Control, no MAGEG1. (B) Co-immunoprecipitation from HEK293 cells co-transfected with S-tagged wild-type and/or mutant MAGEG1 and with FLAG-tagged NSE4b. Lysates were immunoprecipitated with protein-S and immunoblotted with either S-HRP (top) or anti-FLAG (bottom). (C) Structure of the C-terminal domain of MAGEG1 (aa 175 to 270) [14] with the NSE4b-interacting residues indicated in red.

MAGEG1 is the mammalian ortholog of Nse3 and there are two orthologs of Nse4, namely NSE4a and NSE4b [18]. Based on our findings with *S. pombe*, we have analysed the interactions between a limited number of mutant MAGEG1 (aa 55 to 292) proteins and NSE4b in our yeast-2-hybrid system. Six mutants that change conserved hydrophobic residues in MAGEG1 within its helices H4 (M180, I181, L185) or H8 (F266, V267, V270) (Supplementary Table S2) disrupted this interaction (Figure 5A, row 2). To obtain further support for the disruptive effect of these mutations, we have expressed three of the MAGEG1 mutants (full-length) together with NSE4b in HEK293 cells and examined their interactions with NSE4b by immunoprecipitation from cell extracts (Figure 5B). With two of these mutants the interaction was clearly reduced (lanes 9 and 10) and there was a modest reduction with the third mutant (lane 8). The positions of the mutated amino acids on the structure of MAGEG1 are shown in Figure 5C. These data are in accordance with the *S. pombe* findings (Figure 2B) and demonstrate the evolutionary conservation of the Nse3-Nse4 binding.

### MAGEG1-NSE4b effects on transcriptional activation

**Figure 6:**
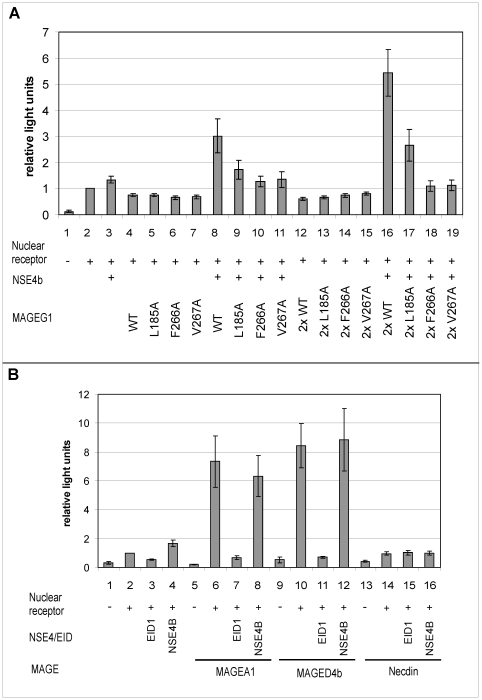
Interplay between MAGE and EID proteins in transcription activation system. (A) Effect of transfected FLAG-NSE4b and S-tagged MAGEG1 on transcriptional activation by SF-1 in HEK293 cells. “2×” indicates twice the concentration of MAGEG1 plasmid used in transfections. (B) Effects of FLAG-tagged EID1 or NSE4b on transcriptional activation by different MAGE proteins. The reporter activity in each column is normalised to the activity with nuclear receptor but with neither MAGE nor EID (column 2). Results show mean ± SEM of 3–5 independent transfections.

NSE4b/EID3 was first identified as a member of the EID (E1A-like inhibitor of differentiation) family of transcriptional repressors and was shown to inhibit transcriptional activation from several promoters in HuH7 human hepatoma cells [20]. We were interested to see if the interaction between NSE4b and MAGEG1 might affect transcriptional activation, and to examine this, we used the Gal4-SF1 promoter system to study SF-1 mediated transcription activation [20]. Figure 6A confirms that, in HEK293 cells, nuclear receptor stimulates reporter activity some 5–10-fold (columns 1 and 2). Neither NSE4b nor MAGEG1 had much effect (columns 3 and 4), but there was a dramatic concentration-dependent stimulation of transcription activation when MAGEG1 and NSE4b were expressed together at two different concentrations of MAGEG1 (columns 8 and 16). To confirm that this transcriptional co-activation was the result of a functional interaction between MAGEG1 and NSE4b, we co-transfected the cells with NSE4b and the series of mutants of MAGEG1 that reduced or abolished its interaction with NSE4b (see Figure 5). As seen in Figure 6A, lanes 9–11 and lanes 17–19, transcriptional activation by the mutant MAGEG1 proteins and NSE4b was much less than with the corresponding concentration of wild-type MAGEG1 (Lanes 8 and 16).

### Evolutionary conservation of interactions between MAGE and EID proteins

The MAGE family consists of a single family member (the ortholog of Nse3) in all eukaryotic organisms except for placental mammals. In contrast, in placental mammals, there are tens of MAGE gene (and pseudogene) copies per genome ([16]; JP unpublished data). For example, the human genome contains 22 class I and 11 class II MAGE genes (Table 2 and Figure 2C). However, we showed previously that only MAGEG1 is found in the SMC5-6 complex and is the true ortholog of Nse3 [18].

**Table 2:** MAGE and EID proteins used in this study.

*S. pombe* protein	Human proteins	
Nse3	MAGEG1	Type II MAGE – ortholog of Nse3
	MAGEF1	Type II MAGE - closely related to MAGEG1
	MAGED4b	Type II MAGE
	Necdin	Type II MAGE – expressed in postmitotic neurons
	MAGEA1	Type I MAGE
Nse4	EID1	Identified as transcriptional repressor
	EID2	Identified by sequence similarity to EID1. Also shown to be transcriptional repressor
	EID2b	Identified by sequence similarity to EID2. Also shown to be transcriptional repressor
	EID3/NSE4b	Identified by sequence similarity to EID1. Testis-specific transcriptional repressor. Ortholog of Nse4 in testis.
	NSE4a	Ortholog of Nse4 in somatic cells

**Figure 7:**
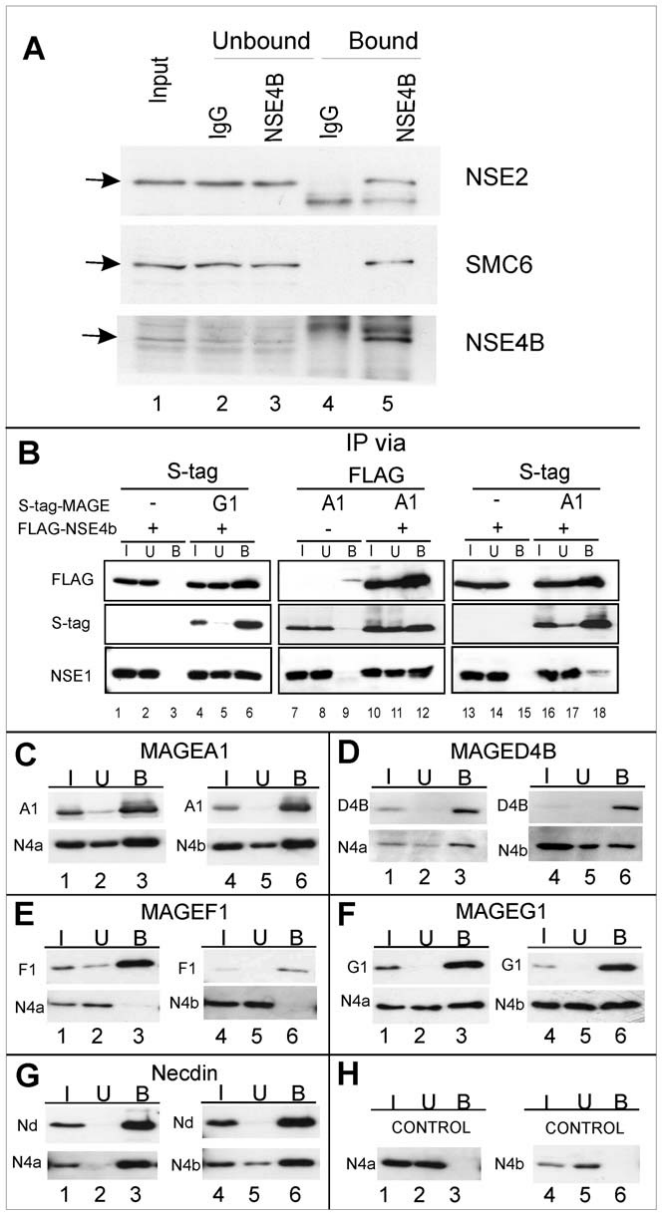
Binding of different MAGE proteins to NSE4a and NSE4b/EID3 proteins. (A) Cell-free extracts of mouse testes were immunoprecipitated with either IgG or anti-NSE4b followed by Western blotting with the indicated antibodies. (B) S-tagged MAGEA1 or MAGEG1 were co-expressed with FLAG-tagged NSE4b in HEK293 cells. In lanes 1–6 and 13–18, extracts were precipitated with S-protein, whereas in lanes 7–12 they were immunoprecipitated with anti-FLAG antibody. (C–H) S-tagged Class I MAGE protein A1 (C), and class II MAGE proteins D4b (D), F1 (E), G1 (F), necdin (G) or vector alone (H) were co-transfected with FLAG-tagged NSE4a (N4a) (lanes 1–3) or NSE4b/EID3 (N4b) (lanes 4–6). Extracts were immunoprecipitated with S-protein and Western blotted with S-HRP and anti-FLAG antibody.

Of the two orthologs of Nse4, only NSE4a was detected in the SMC5-6 complex from cultured cells, but we showed that, when overexpressed following transfection, either paralog could be incorporated into the complex [18]. Examination of EST libraries suggested that NSE4b was expressed mainly in the testis and tissue-specific micro-array data show that, in the mouse, it is expressed exclusively in testis (http://biogps.gnf.org/#goto=genereport&id=493861). This raised the possibility that it might be the SMC5-6 kleisin in the testis. To test this directly, we used antibodies against NSE4b for immunoprecipitations from extracts of mouse testes. The immunoprecipitates were analysed for other components of the SMC5-6 complex by immunoblotting with anti-hSMC6 and anti-hNSE2/MMS21. Figure 7A, lane 5, shows that both mSMC6 and mNSE2/mMMS21 were co-immunoprecipitated. Finally we immunoprecipitated SMC6 from mouse testes and analysed the immunoprecipitates by mass spectrometry. NSE4b (as well as NSE4a) and other expected members of the complex were detected (data not shown), confirming that NSE4b is a testis-specific component of SMC5-6.

In Figure 7B (lane 6), we confirm that, when expressed in HEK293 cells, immunoprecipitation of MAGEG1 coprecipitates both NSE4b and NSE1 [18]. However the hydrophobic character of the Nse4-interacting residues in Nse3/MAGEG1 is well conserved in the sequences of all the human MAGE proteins (Figure 2C), suggesting that the Nse3-Nse4 interaction might be conserved more widely. We previously showed that FLAG-tagged NSE4b could interact with NSE1 and SMC6, presumably as part of the SMC5-6 complex [18]. The immunoprecipitation shown in Figure 7B (lane 12, bottom panel) confirms the interaction of NSE4b with NSE1, but also shows an interaction with MAGEA1 (lane 12, middle panel). When we did the immunoprecipitation the other way round, immunoprecipitating MAGEA1, the interaction with NSE4b was confirmed (lane 18, top panel), but there was minimal interaction with NSE1 (lane 18, bottom panel), suggesting that the NSE4b-MAGEA1 formed a complex that was separate from the SMC5-6 complex. To extend these findings, we have co-expressed representative S-tagged MAGE proteins with FLAG-tagged NSE4a or b and analysed the interactions by co-immunoprecipitation. The results are shown in Figures 7C–H. Interestingly most of the MAGE proteins tested interacted significantly with both NSE4 paralogs (lanes 3 and 6, lower panels in each figure section). Figure 7C–H show clear interaction of both paralogs with the Type I MAGE A1 (C) and the type II MAGE D4b (D) and necdin (G). These can be compared with the previously described interactions of MAGEG1 with NSE4a and b (Figure 7F and [18]), which are known to be components of the SMC5-6 complex. An exception is MAGEF1, which does not appear to interact with either paralog (Figure 7E).

**Figure 8:**
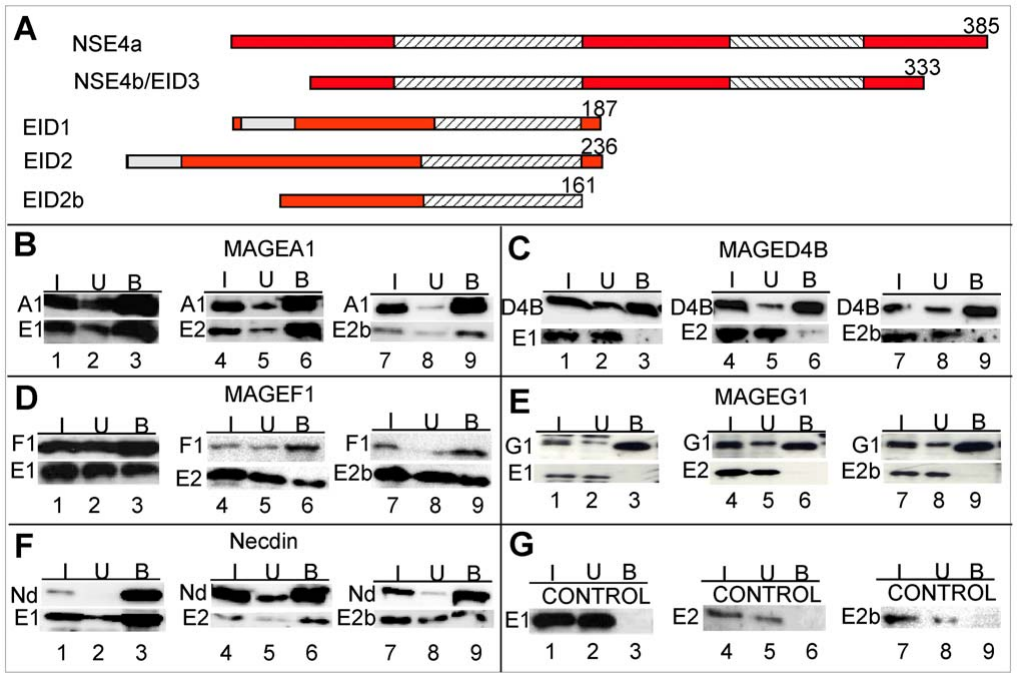
Interactions of MAGE proteins with EID1, 2 and 2b. (A) Alignments of the five members of the human EID family. Hatched and grey boxes indicate kleisin motifs and regions of homology between EID1 and 2, respectively. (B–G) S-tagged Class I MAGE protein A1 (B), and class II MAGE proteins D4b (C), F1 (D), G1 (E), necdin (F) or vector alone (G) were co-transfected with FLAG-tagged EID1 (E1) (lanes 1–3), EID2 (E2) (lanes 4–6) or EID2b (E2b) (lanes 7–9) into HEK293. Extracts were immunoprecipitated with S-protein and Western blotted with S-HRP and anti-FLAG antibody.

**Figure 9:**
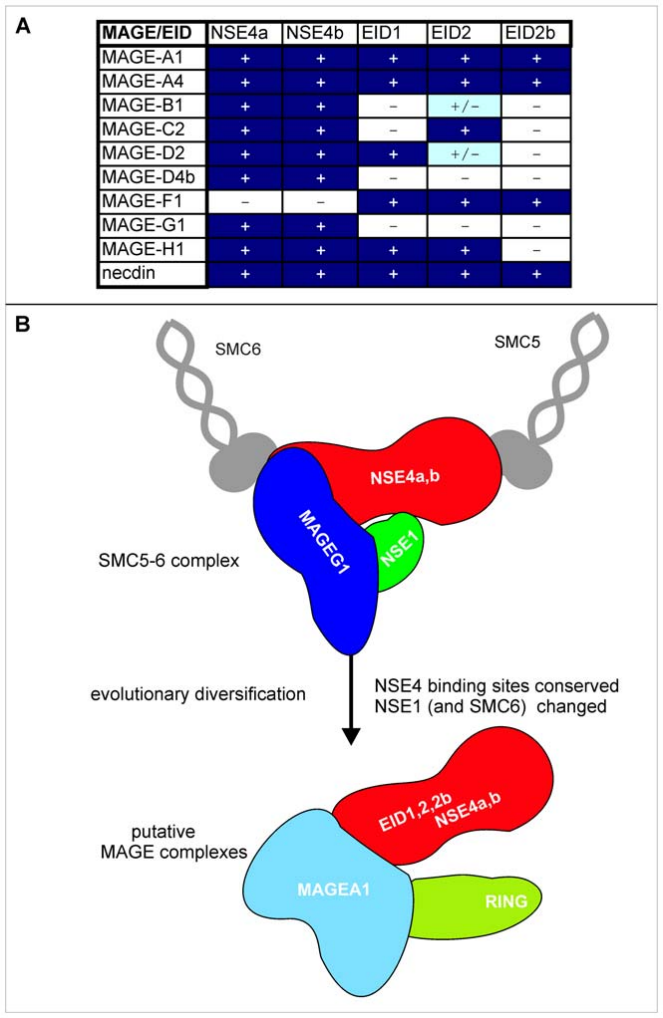
Interactions of MAGE proteins. (A) Data from co-immunoprecipitation experiments were analysed visually; + and − signify whether or not an interaction was detected. (B) Cartoon of MAGE interactions showing evolutionary diversification of hypothetical MAGE complexes.

The N-terminal part of yeast Nse4 mediates the interaction with Nse3 (Fig. 1C). Interestingly, NSE4a and NSE4b/EID3 are members of another gene family, the EID family, whose other members, namely EID1, 2 and 2b (Table 2), have substantial sequence identity to the N-terminal part of the Nse4 proteins (Figure 8A and [20]). Interactions of S-tagged MAGE proteins co-expressed with FLAG-tagged EID1, 2 and 2b in HEK293 cells are shown in Figure 8B–G. As with the NSE4 paralogs (Figure 7), we found that the MAGE proteins interacted with the EID proteins, albeit to different extents. Interestingly MAGEG1 did not interact with any of the EID proteins (Figure 8E, lanes 3, 6, 9) while MAGEF1 precipitated all of them (Fig. 8D, lanes 3, 6, 9). Because of different levels of expression of the different MAGE proteins, it is not possible to make quantitative comparisons, but a summary of all the interactions that we have analysed is presented in Figure 9A.

### Effects of MAGE and EID proteins on gene expression

When we analysed the effect of several different MAGE proteins in the transcription activation system, MAGEA1 and MAGED4b had large stimulatory effects on Gal4-SF1 activity in HEK293 cells (Fig. 6B, columns 6 and 10), which were completely abolished by EID1 (columns 7 and 11) but were unaffected by NSE4b (columns 8 and 12). Necdin alone had little transactivation activity (column 14), but, in its presence, the reporter activity was resistant to inhibition by EID1 (column 15), (in keeping with the findings of Bush and Wevrick [21]), and NSE4b also had little effect (column 16). None of the MAGE/EID interactions resulted in transcriptional co-activation as found between MAGEG1 and NSE4b (Fig. 6A).

## Discussion

In our previous studies we showed that Nse1, Nse3 and Nse4 formed a sub-complex within the highly conserved SMC5-6 protein complex and that Nse3 was structurally homologous to the MAGE protein family [8]. We have now refined the architectural definition of this sub-complex and focussed on the Nse3/MAGE protein. We have identified a surface on Nse3 that interacts with Nse4 and a structural domain of Nse3 that interacts with Nse1. This analysis is based on modelling the structure of Nse3 onto the structure of MAGEA4 and G1 deposited in the Protein Database. The validity of our conclusions obviously depends on the accuracy of our modelling. The high level of sequence similarity between MAGE proteins and Nse3 together with the internal self-consistency of our observations gives us confidence that our modelling is reasonably accurate. The interacting region between *S. pombe* Nse1 and Nse3 that we have defined, based on our two-hybrid and modelling analysis, corresponds well with that deduced from the crystal structure of the orthologous human NSE1-MAGEG1 [14]. Furthermore NSE1 and the hydrophobic cleft on Nse3/MAGEG1 that we predict forms the interaction surface with Nse4 are positioned on the same face of Nse3/MAGEG1. We predict that Nse1/NSE1 and the hydrophobic cleft together form a pocket in which the N-terminus of Nse4/NSE4a/4b is located, as shown schematically in Figure 1F and 9B.

We have expanded our findings into mammalian systems. We showed previously that there were two NSE4 paralogs in mammals [18]. Using yeast 2-hybrid analysis and co-immunoprecipitation, we have demonstrated that mutations in MAGEG1 corresponding to those that reduced the interaction with Nse4 in *S. pombe*, also reduced the interaction of MAGEG1 with NSE4b. To gain further insights into the functional significance of the NSE4b/MAGEG1 interaction, we used a transcription activation reporter system. Intriguingly, there was a synergistic interaction on transcription activation between MAGEG1 and NSE4b (though not between MAGEG1 and NSE4a – unpublished data), and it was reduced in MAGEG1 mutants that diminished the interaction between MAGEG1 and NSE4b. In our experimental system, we think that this transcriptional activation most likely results from a binary “free” complex of NSE4b and MAGEG1. However it raises the question of whether it can also occur in the context of the SMC5-6 complex. This would indicate a novel role for the SMC5-6 complex in transcriptional activation. Further studies are required to resolve this issue.

The evolutionary diversification of the MAGE protein family is remarkable. There is only a single member in fungi, insects [22], birds [23], fish and plants [24], and its most likely function is as part of the SMC5-6 complex. In non-placental mammals there is one member in platypus and two in opossum. In contrast, in placental mammals, there are 33 (+22 pseudogenes) in man, a similar number in mouse and even more in elephants (JP, unpublished data). We showed previously that MAGEG1 is the only MAGE protein detected in the SMC5-6 complex, and that MAGEF1 could not be integrated into the complex [18]. This is consistent with our finding that MAGEF1 does not interact with NSE4a or b (Figure 7E). Instead MAGEF1 protein can form complexes with EID proteins (which lack the C-terminal WHD domain essential for binding to the SMC5 head domain). Remarkably we found that most of the MAGE proteins that we examined interacted with both NSE4a and NSE4b (Figure 9A). However, with the exception of the MAGEG1 interactions, the MAGE-NSE4 interactions do not take place in the context of the SMC5-6 complex, since neither NSE1 nor SMC6 is found in the immunoprecipitates (Fig. 7B, data not shown). Consistent with our results, Doyle et al. found that most of the MAGE proteins that they examined were unable to interact with NSE1 [14]. We have shown that Nse1 stabilizes the interaction between *S. pombe* Nse4 and Nse3 (Figure 4A, [8]), and the same is probably the case for the human orthologs. Without NSE1, it is likely that the MAGE-NSE4 subcomplexes are not able to bind to the SMC6-SMC5-NSE2 subcomplex (Fig. 9B). Furthermore, we previously showed that not only Nse4 but also Nse3 (as well as Nse5 and Nse6 in *S. pombe*) bound to the head domain of Smc6 ([13]; K. Bednarova unpublished data). We speculate that the MAGE proteins (other than MAGEG1), have lost their ability to bind to the SMC6 head domain and to NSE1. The evolutionary diversification of such a binding surface(s) then resulted in a gain of new binding partners and the formation of novel MAGE complexes with RING-finger proteins (Figure 9B) ([14]; our unpublished data).

Remarkably, the EID family shows a similar pattern of evolutionary diversification to the MAGE family, albeit to a less dramatic extent, namely a single member (Nse4) in most eukaryotes up to non-placental mammals (although there are two in the plant *Arabidopsis thaliana*; [24]) and four members in placental mammals. The fifth member, EID2b, is found only in rodents and primates. Our finding that, of the pairs that we examined, most MAGE proteins interacted with most of the EID proteins (Figure 9A) suggests that the diversification of these two protein families may be connected.

Interestingly, two tumour-related mutations in MAGE proteins were described recently. In MAGEA1 Glu217 (corresponding to Phe235 in yeast Nse3, Table 1) was mutated to Lys in a melanoma sample [25]. We speculate that this change could disturb the MAGEA1 binding to NSE4/EID partners. Similarly in MAGEC1 Ile1001 (corresponding to Met214 in yeast Nse3, Table 1) was mutated to Phe in glioblastoma multiforme cells [26]. Although this change is less severe, it could change the affinity and/or specificity of the binding of MAGEC1 to its putative NSE4/EID partner.

The physical interaction between the MAGE and EID proteins raises the question of their functional significance. In contrast to the broadly similar physical interactions between members of the two families, their effects in the transcriptional activation reporter system were quite different. In the EID family, only EID1 repressed transcription in the Gal4-SF1 system in HEK293 cells. Of the MAGE proteins examined, MAGEA1 and D4b were strong transcription co-activators, whereas several other MAGE proteins had little effect. There are various reports in the literature on the effects of MAGE proteins on transcription systems. MAGEA1 represses transcription mediated by Ski interacting protein [27], whereas Wilson and co-workers reported that MAGEA11 increased the transcriptional activity of the androgen receptor [28] via an interaction with p300 [29]. MAGED1 was shown to be a co-activator of the RORα and RORγ proteins, but this co-activation did not require the MHD of MAGED1 [30]. We found that, when EID and MAGE proteins were co-expressed, EID1 reversed the co-activation mediated by MAGEA1 and MAGED4b, whereas it had no effect on the much lower activation in the presence of necdin. The latter result agrees with the finding of Bush and Wevrick [21]. Our results suggest a relatively specific functional interplay between MAGE and EID proteins which contrasts with the general physical interactions that we have observed. It is evident that other proteins interacting with these partners may influence the transcription level. Much more detailed studies need to be carried out in future work in order to unravel the nature of these complex interactions and to understand the functions of these two protein families in their normal cellular contexts.

In conclusion, we have found that, despite the evolutionary diversification of the MAGE family, the characteristic hydrophobic surface shared by all MAGE proteins from yeast to humans mediates its binding to NSE4/EID proteins.

## Materials and Methods

### Plasmids

All pTriEx4 plasmids described in this study were generated by PCR and ligase-independent cloning (Merck). The primers used for PCR amplification are listed in Supplementary Table S3. To get pGBKT7-MAGEG1(aa55-292) construct NcoI-XhoI fragment of pTriEx4-MAGEG1(aa55-290) clone was inserted into the pGBKT7 vector digested with NcoI-SalI restriction enzymes. EcoRI-XhoI fragment of pTriEx4-NSE4b(aa1-333) was cloned into pGADT7 yeast-2-hybrid vector. The EID2 ORF was amplified with CTC GAG ATG GCA GAC AGC AGT GTC and TCT AGA CTA TTC TCT ATT GAT AAA C primers and inserted into pGEM-T-easy vector (Promega). The pGEM-EID2 construct was cut with XhoI-XbaI restriction enzymes and subcloned into pCI-neo-FLAG vector. Similarly EID2b ORF was cloned into pGEM-T-easy vector (CTC GAG ATG GCG GAG CCG ACT GGG and ACG CGT TCA GTC GGC CAG AGG AC) and then subcloned into pCI-neo-FLAG vector (using XhoI-MluI restriction enzymes). The other constructs were described previously [8], [13], [18].

### Protein-S pull down assays

His-S tag-fusion protein extracts from *E.coli* strain C41 were preincubated with protein S-agarose beads (Merck). Then *in vitro*-expressed proteins in a total volume of 200 microliter of HEPES buffer were added and incubated overnight [13]. Input, unbound, and bound fractions were separated by SDS-PAGE, transferred to nitrocellulose membranes, and analyzed by phosphorimaging and immunoblotting with anti-His antibody (Sigma).

### Nse3 structure modeling

The crystal structures of MAGE A4 and G1 (PDB entries 2WA0 and 3NW0) were used as the input structures for Nse3 (aa 90 to 310). The I-TASSER server [31] was used to model the Nse3 structure.

### Site-directed mutagenesis

The QuikChange II XL system (Stratagene) was used to create point mutations in the pGBKT7-Nse3(aa1-328), pGBKT7-MAGEG1(aa55-292) and pTriEx4-MAGEG1(aa1-304) plasmids; the primers are listed in Supplementary Tables S1 and S2.

### Yeast hybrid assays

The Gal4-based two-hybrid system was used to analyze Nse3 mutants. Each pGBKT7-Nse3(aa1-328) mutant was cotransformed either with pOAD-Nse1(aa1-223) or pACT2-Nse4(aa1-300) construct. Similarly, pGBKT7-MAGEG1(aa55-292) mutants were cotransformed either with pOAD-hNSE1(aa1-266) or pGADT7-hNSE4b(aa1-333) plasmid. Colonies were inoculated into YPD media and cultivated overnight. 10- and 100-fold dilutions were dropped onto the SD-Leu,-Trp (control) and SD-Leu, -Trp, -His (with 0, 1, 2, 5, 10, 15, 20, 30, 60, 120 mM 3-aminotriazole) plates. Each mutant was cotransformed at least twice into *S. cerevisiae* MaV203 yeast strain and at least two independent drop tests were carried out from each transformation. In addition, the results and mutant expression levels were verified in another *S. cerevisiae* Y190 yeast two-hybrid strain. For yeast-3-hybrid tests, three plasmids pGBKT7-Nse3(aa1-328), pACT2-Nse4(aa1-300) and pPM587-Nse1(aa1-232) were cotransformed into PJ69-4a yeast strain and selected on SD-Leu, -Trp, -Ura plates. Drop tests were carried out on SD-Leu, -Trp, -Ura, -His (with 0, 1, 2, 5, 10, 15, 20, 30, 60, 120 mM 3-aminotriazole) plates at 30°C.

### Generation of Nse3 mutant strains of *S. pombe*

The *Nse3* mutant strains were created using Cre recombinase-mediated cassette exchange, as detailed in Watson *et al.* [32]. The *S. pombe* strain ‘501’ (*ura4-D18, leu1-32, ade6-704, h−*) was used to construct the Nse3 base strain with the loxP site integrated 198 bp upstream and the *ura4+* marker and loxM3 site integrated 98 bp downstream of the *nse3* ORF. A fragment comprising the *nse3* ORF, as well as the 198 bp upstream and 98 bp downstream sequences were amplified and cloned into SpeI and SphI sites of pAW6. Site-directed mutagenesis was carried out using QuikChange Kit (Stratagene). Mutated sequences flanked by loxP and loxM3 sites were then cloned into pAW7 (*LEU2* ^+^ marker) and transformed into the *Nse3* base strain. *ura^+^*, *leu^+^* transformants were selected in the presence of thiamine (i.e. in absence of Cre recombinase expression), grown in nonselective medium for 24 hours, and then plated onto medium containing 5-fluoroorotic acid to select clones in which cassette exchange took place. 5-FOA^R^ and *leu^−^* colonies were picked and the presence of the respective mutations verified by sequencing.

### Spot tests for sensitivity to DNA damaging agents

*S. pombe* cultures were grown to mid log phase, concentrated to 3×10^7^ cells/ml, and serial 6-fold dilutions were spotted onto rich media with or without the indicated dose of DNA-damaging agents. Subsequently, plates were incubated at the indicated temperature for 3–4 days.

### Mammalian cell culture and luciferase assays

HEK293 cells (DSMZ, Germany) were grown in DMEM supplemented with 10% foetal bovine serum and 100 µg/ml penicillin/streptomycin (Invitrogen). Plasmid transfections were carried out using calcium phosphate precipitation. For luciferase assays, cells transfected with pUAS-tk-luc [33] and pHRL-CMV (Promega) and with or without combinations of pSG4-Gal4-mSF-1-N1 [20], EID and MAGE constructs were processed and luciferase activity determined using the dual luciferase assay kit according to the manufacturer's instructions (Promega).

### Antibodies

Full-length hNSE4b was expressed in bacteria as a glutathione S-transferase fusion, purified on glutathione Sepharose (GE Healthcare) according to the manufacturer's instructions, and used to inoculate two rabbits for antibody production (Eurogentec). Antibodies were affinity purified using antigen immobilized with Aminolink Plus coupling gel (Pierce). hNSE1, hNSE2 and hSMC6 antibodies have been described previously [18]. Anti-FLAG M2 (Sigma) and S-HRP (Merck) commercial antibodies were also used in this study.

### Immunoprecipitations

Lysates were made from transfected HEK293 cells by scraping in lysis buffer (50 mM Tris-HCl pH7.5, 0.5% NP40, 40 mM NaCl, 2 mM MgCl_2_, 1× protease inhibitor cocktail [Roche], 1 mM NEM, 25 U/ml benzonase [Merck]). Lysates were incubated for 30 minutes on ice, cleared by centrifugation at 13000 rpm for 10 minutes and the NaCl concentration adjusted to 150 mM. Agarose beads conjugated to S protein (Merck) or anti-FLAG antibody (Sigma) were mixed with lysates for 4 hours at 4°C. Beads were washed 3 times with wash buffer (50 mM Tris-HCl pH7.5, 150 mM NaCl, protease inhibitors) before beads were resuspended in SDS loading buffer.

Lysates for testes were prepared by addition of lysis buffer (50 mM Tris pH7.5, 1% triton, 40 mM NaCl, 2 mM MgCl_2_, 2× protease inhibitor cocktail [Roche], 1 mM NEM, 25 U/ml benzonase) and 20 strokes with a loose Dounce homogenizer, followed by treatment as above. Lysates were depleted of non-specific binding proteins by incubation with beads cross-linked to rabbit IgG for 1 hour, followed by incubation with the desired antibody for 2 h at 4°C. They were then centrifuged at 15000 rpm for 10 minutes and the supernatant added to protein G beads. Following mixing for 1 h at 4°C, samples were washed and processed as above.

For immunoprecipitations performed for mass spectrometry, lysates were prepared as above, but were then incubated with antibodies (anti-SMC6 or IgG) cross-linked to protein A-agarose beads [18]. Following immunoprecipitation, samples were washed as previously, followed by 3 washes in low Tris buffer (4 mM Tris-HCl pH 7.5, 150 mM NaCl, protease inhibitors). Elution was performed by incubation with 200 mM glycine pH 2.5 for 5 min at room temperature. This sample was then neutralised by adding 1/10^th^ volume of 1 M Tris-HCl pH 8.8. Following reduction and alkylation of samples, proteins were digested with 2.5 ng/µl trypsin for 16 h at 37°C. The resulting peptide mixture was diluted in 0.1% trifluoroacetic acid for analysis by nano-liquid chromatography-tandem mass spectrometry at the Sussex Centre for Proteomics using an LTQ-Orbitrap FT-MS (Thermo Fisher). Tandem mass spectra were extracted by Bioworks version v.3.3 (Thermo Fisher Scientific), and all MS/MS samples were analyzed using Sequest (Thermo Fisher Scientific version SRF v. 5) which was set up to search the ipi.MOUSE.v.3.55 database (55956 entries) with a fragment ion mass tolerance of 1.0 Da and a parent ion tolerance of 5.0 ppm. Deamidation of asparagine, oxidation of methionine and iodoacetamide derivative of cysteine were specified as variable modifications.

Scaffold v.3.00.03 (Proteome Software Inc.) was subsequently used to validate the MS/MS based identifications. Peptide identifications were accepted if they could be established at greater than 95.0% probability as specified by the Peptide Prophet algorithm [34], and protein identifications were accepted if they could be established at greater than 99.0% probability and contained at least 2 identified peptides. Protein probabilities were assigned by the Protein Prophet algorithm [35].

## Supporting Information

Table S1 **Primers used for site-directed mutagenesis of** ***S. pombe*** **Nse3.**
(DOC)

Table S2 **Primers used for site-directed mutagenesis of human MAGEG1.**
(DOC)

Table S3 **Primers used for pTriEx4 constructs.**
(DOC)
