# Supplementary material for: Interactions between the Nse3 and Nse4 Components of the SMC5-6 Complex Identify Evolutionarily Conserved Interactions between MAGE and EID Families
Source: PLoS One. 2011 Feb 25;6(2):e17270. doi: 10.1371/journal.pone.0017270 (PMC3045436; doi:10.1371/journal.pone.0017270)
Supplement: Table S1 — Primers used for site-directed mutagenesis of S. pombe Nse3. (DOC) [file pone.0017270.s001.doc]

**Table S1. Primers used for site-directed mutagenesis of *S. pombe* Nse3**

| **Mutation** | **Primer (forward / reverse)** |
| --- | --- |
| **L93A** | C GCA ATA AAT TTT CAA CTA gcG GTA CGA AAT GTG GTC CG |
|  | CG GAC CAC ATT TCG TAC Cgc TAG TTG AAA ATT TAT TGC G |
| **V94A** | C GCA ATA AAT TTT CAA CTA TTG GcA CGA AAT GTG GTC CGT TAT GC |
|  | GC ATA ACG GAC CAC ATT TCG TgC CAA TAG TTG AAA ATT TAT TGC G |
| **R95A** | TTT CAA CTA TTG GTA gcA AAT GTG GTC CGT TAT GC |
|  | GC ATA ACG GAC CAC ATT Tgc TAC CAA TAG TTG AAA |
| **V98A** | CTA TTG GTA CGA AAT GTG GcC CGT TAT GCA ATA TGC TCC C |
|  | G GGA GCA TAT TGC ATA ACG GgC CAC ATT TCG TAC CAA TAG |
| **R99A** | G GTA CGA AAT GTG GTC gcA TAT GCA ATA TGC TCC C |
|  | G GGA GCA TAT TGC ATA Tgc GAC CAC ATT TCG TAC C |
| **Y100A** | GTA CGA AAT GTG GTC CGT gcT GCA ATA TGC TCC CAA AC |
|  | GT TTG GGA GCA TAT TGC Agc ACG GAC CAC ATT TCG TAC |
| **I102A** | GTG GTC CGT TAT GCA gcA TGC TCC CAA ACT TCT C |
|  | G AGA AGT TTG GGA GCA Tgc TGC ATA ACG GAC CAC |
| **Q105A** | C CGT TAT GCA ATA TGC TCC gcA ACT TCT CAT AAC ACA ATT ACA CG |
|  | CG TGT AAT TGT GTT ATG AGA AGT Tgc GGA GCA TAT TGC ATA ACG G |
| **S107A** | GCA ATA TGC TCC CAA ACT gCT CAT AAC ACA ATT ACA CG |
|  | CG TGT AAT TGT GTT ATG AGc AGT TTG GGA GCA TAT TGC |
| **H108A** | GCA ATA TGC TCC CAA ACT TCT gcT AAC ACA ATT ACA CGA AAA GAC |
|  | GTC TTT TCG TGT AAT TGT GTT Agc AGA AGT TTG GGA GCA TAT TGC |
| **N109A** | GC TCC CAA ACT TCT CAT gcC ACA ATT ACA CGA AAA GAC |
|  | GTC TT T TCG TGT AAT TGT Ggc ATG AGA AGT TTG GGA GC |
| **T112A** | CT TCT CAT AAC ACA ATT gCA CGA AAA GAC ATA GTT C |
|  | G AAC TAT GTC TTT TCG TGc AAT TGT GTT ATG AGA AG |
| **R113A** | CAT AAC ACA ATT ACA gcA AAA GAC ATA GTT CAA AAA GC |
|  | GC TTT TTG AAC TAT GTC TTT Tgc TGT AAT TGT GTT ATG |
| **K114A** | CAT AAC ACA ATT ACA CGA gcA GAC ATA GTT CAA AAA GC |
|  | GC TTT TTG AAC TAT GTC Tgc TCG TGT AAT TGT GTT ATG |
| **K119A** | CGA AAA GAC ATA GTT CAA gcA GCA TTC CCT GAA GGT AC |
|  | GT ACC TTC AGG GAA TGC Tgc TTG AAC TCT GTC TTT TCG |
| **F121A** | GAC ATA GTT CAA AAA GCA gcC CCT GAA GGT ACA TCC CG |
|  | CG GGA TGT ACC TTC AGG Ggc TGC TTT TTG AAC TAT GTC |
| **E123A** | GTT CAA AAA GCA TTC CCT GcA GGT ACA TCC CGA AAT C |
|  | G ATT TCG GGA TGT ACC TgC AGG GAA TGC TTT TTG AAC |
| **T125A** | CAA AAA GCA TTC CCT GAA GGT gCA TCC CGA AAT CTT TTT CAA TCC G |
|  | C GGA TTG AAA AAG ATT TCG GGA TGc ACC TTC AGG GAA TGC TTT TTG |
| **R127A** | C CCT GAA GGT ACA TCC gcA AAT CTT TTT CAA TCC G |
|  | C GGA TTG AAA AAG ATT Tgc GGA TGT ACC TTC AGG G |
| **F130A** | GT ACA TCC CGA AAT CTT gcT CAA TCC GTT TTT GAA G |
|  | C TTC AAA AAC GGA TTG Agc AAG ATT TCG GGA TGT AC |
| **Q131A** | GGT ACA TCC CGA AAT CTT TTT gcA TCC GTT TTT GAA GAA GC |
|  | GC TTC TTC AAA AAC GGA Tgc AAA AAG ATT TCG GGA TGT ACC |
| **V133A** | CC CGA AAT CTT TTT CAA TCC GcT TTT GAA GAA GCA GAT CG |
|  | CG ATC TGC TTC TTC AAA AgC GGA TTG AAA AAG ATT TCG GG |
| **F134A** | CTT TTT CAA TCC GTT gcT GAA GAA GCA GAT CGA |
|  | TCG ATC TGC TTC TTC Agc AAC GGA TTG AAA AAG |
| **E135A** | CTT TTT CAA TCC GTT TTT GcA GAA GCA GAT CGA CAG |
|  | CTG TCG ATC TGC TTC TgC AAA AAC GGA TTG AAA AAG |
| **E136A** | CAA TCC GTT TTT GAA GcA GCA GAT CGA CAG TTG CAG C |
|  | G CTG CAA CTG TCG ATC TGC TgC TTC AAA AAC GGA TTG |
| **A137K** | CC GTT TTT GAA GAA aaA GAT CGA CAG TTG CAG C |
|  | G CTG CAA CTG TCG ATC Ttt TTC TTC AAA AAC GG |
| **R139A** | CC GTT TTT GAA GAA GCA GAT gcA CAG TTG CAG CTA AGC |
|  | GCT TAG CTG CAA CTG Tgc ATC TGC TTC TTC AAA AAC GG |
| **Q140A** | C GTT TTT GAA GAA GCA GAT CGA gcG TTG CAG CTA AGC TTT GGG TTT CG |
|  | CG AAA CCC AAA GCT TAG CTG CAA Cgc TCG ATC TGC TTC TTC AAA AAC G |
| **L141A** | GAA GAA GCA GAT CGA CAG gcG CAG CTA AGC TTT GGG TTT CG |
|  | CG AAA CCC AAA GCT TAG CTG Cgc CTG TCG ATC TGC TTC TTC |
| **S144A** | GAT CGA CAG TTG CAG CTA gcC TTT GGG TTT CGT CTC GTC |
|  | GAC GAG ACG AAA CCC AAA Ggc TAG CTG CAA CTG TCG ATC |
| **F145A** | CGA CAG TTG CAG CTA AGC gcT GGG TTT CGT CTC GTC GCG |
|  | CGC GAC GAG ACG AAA CCC Agc GCT TAG CTG CAA CTG TCG |
| **G146A** | CAG TTG CAG CTA AGC TTT GcG TTT CGT CTC GTC GCG |
|  | CGC GAC GAG ACG AAA CgC AAA GCT TAG CTG CAA CTG |
| **F147A** | G TTG CAG CTA AGC TTT GGG gcT CGT CTC GTC GCG GTT ACA C |
|  | G TGT AAC CGC GAC GAG ACG Agc CCC AAA GCT TAG CTG CAA C |
| **L149A** | G CTA AGC TTT GGG TTT CGT gcC GTC GCG GTT ACA CAA TCG |
|  | CGA TTG TGT AAC CGC GAC Ggc ACG AAA CCC AAA GCT TAG C |
| **V152A** | C TTT GGG TTT CGT CTC GTC GCG GcT ACA CAA TCG AAT CGG |
|  | CCG ATT CGA TTG TGT AgC CGC GAC GAG ACG AAA CCC AAA G |
| **S155A** | GT CTC GTC GCG GTT ACA CAA gCG AAT CGG AAA AAA GAC ATG GCG |
|  | CGC CAT GTC TTT TTT CCG ATT CGc TTG TGT AAC CGC GAC GAG AC |
| **H180A** | CG AAT GCC AAT TCA TCA AAT CTA gcT AGA TAC TGG GTG TTG AGG |
|  | CCT CAA CAC CCA GTA TCT Agc TAG ATT TGA TGA ATT GGC ATT CG |
| **Y182A** | CA TCA AAT CTA CAT AGA gcC TGG GTG TTG AGG TCT ACC |
|  | GGT AGA CCT CAA CAC CCA Ggc TCT ATG TAG ATT TGA TG |
| **V184A** | CA AAT CTA CAT AGA TAC TGG GcG TTG AGG TCT ACC CTT CCT ATG G |
|  | C CAT AGG AAG GGT AGA CCT CAA CgC CCA GTA TCT ATG TAG ATT TG |
| **L185A** | CAT AGA TAC TGG GTG gcG AGG TCT ACC CTT CC |
|  | GG AAG GGT AGA CCT Cgc CAC CCA GTA TCT ATG |
| **T188A** | C TGG GTG TTG AGG TCT gCC CTT CCT ATG GAA CTT C |
|  | G AAG TTC CAT AGG AAG GGc AGA CCT CAA CAC CCA G |
| **L199A** | G GAA CTT CAA AAG GAT TCT CGC gcA ATT GTA GAT TCG GTA TTG G |
|  | C CAA TAC CGA ATC TAC AAT Tgc GCG AGA ATC CTT TTG AAG TTC C |
| **F212A** | G GAT ACT GCT TAT TAT GGA gcT TTG ATG ACT GTG ATC GC |
|  | GC GAT CAC AGT CAT CAA Agc TCC ATA ATA AGC AGT ATC C |
| **M214A** | CT GCT TAT TAT GGA TTT TTG gcG ACT GTG ATC GCA TTC ATA G |
|  | C TAT GAA TGC GAT CAC AGT Cgc CAA AAA TCC ATA ATA AGC AG |
| **T215A** | TAT GGA TTT TTG ATG gCT GTG ATC GCA TTC ATA GC |
|  | GC TAT GAA TGC GAT CAC AGc CAT CAA AAA TCC ATA |
| **V216A** | GGA TTT TTG ATG ACT GcG ATC GCA TTC ATA GCC G |
|  | C GGC TAT GAA TGC GAT CgC AGT CAT CAA AAA TCC |
| **I217A** | GGA TTT TTG ATG ACT GTG gcC GCA TTC ATA GCC GTT TCT C |
|  | G AGA AAC GGC TAT GAA TGC Ggc CAC AGT CAT CAA AAA TCC |
| **A218G** | GGA TTT TTG ATG ACT GTG ATC GgA TTC ATA GCC GTT TCT C |
|  | G AGA AAC GGC TAT GAA TcC GAT CAC AGT CAT CAA AAA TCC |
| **F219A** | G ATG ACT GTG ATC GCA gcC ATA GCC GTT TCT CAT TGC |
|  | GCA ATG AGA AAC GGC TAT Ggc TGC GAT CAC AGT CAT C |
| **I220A** | G ATG ACT GTG ATC GCA TTC gcA GCC GTT TCT CAT TGC AG |
|  | CT GCA ATG AGA AAC GGC Tgc GAA TGC GAT CAC AGT CAT C |
| **V222A** | G ATC GCA TTC ATA GCC GcT TCT CAT TGC AGT GTA GG |
|  | CC TAC ACT GCA ATG AGA AgC GGC TAT GAA TGC GAT C |
| **S223A** | C GCA TTC ATA GCC GTT gCT CAT TGC AGT GTA GGA C |
|  | G TCC TAC ACT GCA ATG AGc AAC GGC TAT GAA TGC G |
| **V227A** | GCC GTT TCT CAT TGC AGT GcA GGA CAT TCA GAA CTG CAA TC |
|  | GA TTG CAG TTC TGA ATG TCC TgC ACT GCA ATG AGA AAC GGC |
| **H229A** | CAT TGC AGT GTA GGA gcT TCA GAA CTG CAA TCT |
|  | AGA TTG CAG TTC TGA Agc TCC TAC ACT GCA ATG |
| **L232A** | GTA GGA CAT TCA GAA gcG CAA TCT TTT TTG CAG |
|  | CTG CAA AAA AGA TTG Cgc TTC TGA ATG TCC TAC |
| **F235A** | GGA CAT TCA GAA CTG CAA TCT gcT TTG CAG GAA CTT CTA ACA GAG |
|  | CTC TGT TAG AAG TTC CTG CAA Agc AGA TTG CAG TTC TGA ATG TCC |
| **L236A** | CAT TCA GAA CTG CAA TCT TTT gcG CAG GAA CTT CTA ACA GAG G |
|  | C CTC TGT TAG AAG TTC CTG Cgc AAA AGA TTG CAG TTC TGA ATG |
| **E238A** | CTG CAA TCT TTT TTG CAG GcA CTT CTA ACA GAG GAA GAA AC |
|  | GT TTC TTC CTC TGT TAG AAG TgC CTG CAA AAA AGA TTG CAG |
| **L239A** | CTG CAA TCT TTT TTG CAG GAA gcT CTA ACA GAG GAA GAA ACA ACC |
|  | GGT TGT TTC TTC CTC TGT TAG Agc TTC CTG CAA AAA AGA TTG CAG |
| **L240A** | G CAA TCT TTT TTG CAG GAA CTT gcA ACA GAG GAA GAA ACA ACC |
|  | GGT TGT TTC TTC CTC TGT Tgc AAG TTC CTG CAA AAA AGA TTG C |
| **P247A** | CA GAG GAA GAA ACA ACC gCT TTG CAT CTG GAT ATT ACT CGC |
|  | GCG AGT AAT ATC CAG ATG CAA AGc GGT TGT TTC TTC CTC TG |
| **L248A** | GAG GAA GAA ACA ACC CCT gcG CAT CTG GAT ATT ACT CGC |
|  | GCG AGT AAT ATC CAG ATG Cgc AGG GGT TGT TTC TTC CTC |
| **H249A** | GAA GAA ACA ACC CCT TTG gcT CTG GAT ATT ACT CGC |
|  | GCG AGT AAT ATC CAG Agc CAA AGG GGT TGT TTC TTC |
| **I252A** | CCT TTG CAT CTG GAT gcT ACT CGC TCG TTA TCT C |
|  | G AGA TAA CGA GCG AGT Agc ATC CAG ATG CAA AGG |
| **S255A** | G CAT CTG GAT ATT ACT CGC gCG TTA TCT CTT TTA GTT CGC |
|  | GCG AAC TAA AAG AGA TAA CGc GCG AGT AAT ATC CAG ATG C |
| **S257A** | G GAT ATT ACT CGC TCG TTA gCT CTT TTA GTT CGC CAA GGG |
|  | CCC TTG GCG AAC TAA AAG AGc TAA CGA GCG AGT AAT ATC C |
| **L259A** | CT CGC TCG TTA TCT CTT gcA GTT CGC CAA GGG TAT TTA G |
|  | C TAA ATA CCC TTG GCG AAC Tgc AAG AGA TAA CGA GCG AG |
| **V260A** | CGC TCG TTA TCT CTT TTA GcT CGC CAA GGG TAT TTA GAT AG |
|  | CT ATC TAA ATA CCC TTG GCG AgC TAA AAG AGA TAA CGA GCG |
| **R261A** | CGC TCG TTA TCT CTT TTA GTT gcC CAA GGG TAT TTA GAT AGA GTG |
|  | CAC TCT ATC TAA ATA CCC TTG Ggc AAC TAA AAG AGA TAA CGA GCG |
| **Q262A** | CG TTA TCT CTT TTA GTT CGC gcA GGG TAT TTA GAT AGA GTG |
|  | CAC TCT ATC TAA ATA CCC Tgc GCG AAC TAA AAG AGA TAA CG |
| **Y264A** | CT CTT TTA GTT CGC CAA GGG gcT TTA GAT AGA GTG AAA GAT G |
|  | C ATC TTT CAC TCT ATC TAA Agc CCC TTG GCG AAC TAA AAG AG |
| **L265A** | CTT TTA GTT CGC CAA GGG TAT gcA GAT AGA GTG AAA GAT GAT ACC C |
|  | G GGT ATC ATC TTT CAC TCT ATC Tgc ATA CCC TTG GCG AAC TAA AAG |
| **R267A** | GTT CGC CAA GGG TAT TTA GAT gcA GTG AAA GAT GAT ACC CAT AAC C |
|  | G GTT ATG GGT ATC ATC TTT CAC Tgc ATC TAA ATA CCC TTG GCG AAC |
| **F276A** | GAT GAT ACC CAT AAC CAG gcT GTT TAT TAT ATT GGA TCC CG |
|  | CG GGA TCC AAT ATA ATA AAC Agc CTG GTT ATG GGT ATC ATC |
| **Y278A** | CC CAT AAC CAG TTT GTT gcT TAT ATT GGA TCC CGT GCA G |
|  | C TGC ACG GGA TCC AAT ATA Agc AAC AAA CTG GTT ATG GG |
| **Y279A** | CC CAT AAC CAG TTT GTT TAT gcT ATT GGA TCC CGT GCA GTA AC |
|  | GT TAC TGC ACG GGA TCC AAT Agc ATA AAC AAA CTG GTT ATG GG |
| **E287A** | TCC CGT GCA GTA ACT GcA ATT TCT ATT GAA GGC |
|  | GCC TTC AAT AGA AAT TgC AGT TAC TGC ACG GGA |
| **L293A** | GAA ATT TCT ATT GAA GGC gcG AAA TCA TTC GTT ACA GAG |
|  | CTC TGT AAC GAA TGA TTT Cgc GCC TTC AAT AGA AAT TTC |
| **F296A** | GAA GGC TTG AAA TCA gcC GTT ACA GAG TTC TTT CCG |
|  | CGG AAA GAA CTC TGT AAC Ggc TGA TTT CAA GCC TTC |
| **V297A** | GGC TTG AAA TCA TTC GcT ACA GAG TTC TTT CCG G |
|  | C CGG AAA GAA CTC TGT AgC GAA TGA TTT CAA GCC |
| **F300A** | CA TTC GTT ACA GAG gcC TTT CCG GAT TCA GAC |
|  | GTC TGA ATC CGG AAA Ggc CTC TGT AAC GAA TG |
| **F301A** | CA TTC GTT ACA GAG TTC gcT CCG GAT TCA GAC ATT G |
|  | C AAT GTC TGA ATC CGG Agc GAA CTC TGT AAC GAA TG |
