# Supplementary material for: Interactions between the Nse3 and Nse4 Components of the SMC5-6 Complex Identify Evolutionarily Conserved Interactions between MAGE and EID Families
Source: PLoS One. 2011 Feb 25;6(2):e17270. doi: 10.1371/journal.pone.0017270 (PMC3045436; doi:10.1371/journal.pone.0017270)
Supplement: Table S2 — Primers used for site-directed mutagenesis of human MAGEG1. (DOC) [file pone.0017270.s002.doc]

**Table S2. Primers used for site-directed mutagenesis of human** MAGEG1

| **Mutation** | **Primer (forward / reverse)** |
| --- | --- |
| **M180A** | ACT ACG GGC CTC CTG gcG ATC GTC TTA GGG CTC |
|  | GAG CCC TAA GAC GAT Cgc CAG GAG GCC CGT AGT |
| **I181A** | ACG GGC CTC CTG ATG gcC GTC TTA GGG CTC ATC |
|  | GAT GAG CCC TAA GAC Ggc CAT CAG GAG GCC CGT |
| **L185A** | ATG ATC GTC TTA GGG gcC ATC TTT ATG AAG GGC |
|  | GCC CTT CAT AAA GAT Ggc CCC TAA GAC GAT CAT |
| **K189A** | GGG CTC ATC TTT ATG gcG GGC AAC ACC ATC AAG |
|  | CTT GAT GGT GTT GCC Cgc CAT AAA GAT GAG CCC |
| **F266A** | ATG AAA GTT CTT AAG gcT GTG GCC AAG GTC CAT |
|  | ATG GAC CTT GGC CAC Agc CTT AAG AAC TTT CAT |
| **V267A** | AAA GTT CTT AAG TTT GcG GCC AAG GTC CAT AAT |
|  | ATT ATG GAC CTT GGC CgC AAA CTT AAG AAC TTT |
| **V270A** | AAG TTT GTG GCC AAG GcC CAT AAT CAA GAC CCC |
|  | GGG GTC TTG ATT ATG GgC CTT GGC CAC AAA CTT |
