# Supplementary material for: Interactions between the Nse3 and Nse4 Components of the SMC5-6 Complex Identify Evolutionarily Conserved Interactions between MAGE and EID Families
Source: PLoS One. 2011 Feb 25;6(2):e17270. doi: 10.1371/journal.pone.0017270 (PMC3045436; doi:10.1371/journal.pone.0017270)
Supplement: Table S3 — Primers used for pTriEx4 constructs. (DOC) [file pone.0017270.s003.doc]

**Table S3. Primers used for** pTriEx4 constructs

| pTriEx4-Nse1(aa110-232) | GAC GAC GAC AAG ATG AAG TGT GAT GAT TAC CAA |
| --- | --- |
|  | GAG GAG AAG CCC GGT TCA CCA GCG TCC TAT AAC GGT C |
| pTriEx4-Nse1(aa110-180) | GAC GAC GAC AAG ATG AAG TGT GAT GAT TAC CAA |
|  | GAG GAG AAG CCC GGT TCA ATT GCT CTC GTA TTC GTT GTG |
| pTriEx4-Nse1(aa170-232) | GAC GAC GAC AAG ATG GCT TAT TTA CAC AAC GAA TAC |
|  | GAG GAG AAG CCC GGT TCA CCA GCG TCC TAT AAC GGT C |
| pTriEx4-Nse3(aa80-307) | GAC GAC GAC AAG ATG GCC ATG GAA GAA CGG AAT GAA ACA G |
|  | GAG GAG AAG CCC GGT CTC GAG TCA ATC AAT GTC TGA ATC CGG |
| pTriEx4-Nse3(aa80-210) | GAC GAC GAC AAG ATG GCC ATG GAA GAA CGG AAT GAA ACA G |
|  | GAG GAG AAG CCC GGT CTC GAG TCA ATA ATA AGC AGT ATC CAA TAC C |
| pTriEx4-Nse3(aa200-307) | GAC GAC GAC AAG ATG CCC ATG GGG ATT GTA GAT TCG GTA TTG G |
|  | GAG GAG AAG CCC GGT CTC GAG TCA ATC AAT GTC TGA ATC CGG |
| pTriEx4-MAGEA1(aa1-309) | GAC GAC GAC AAG ATG CCC ATG GGG ATG TCT CTT GAG CAG AGG AG |
|  | GAG GAG AAG CCC GGT CTC GAG TCA GAC TCC CTC TTC CTC CTC |
| pTriEx4-MAGEA4(aa100-317) | GAC GAC GAC AAG ATG CCC‘ ATG GAC GCA GAG TCC TTG TTC C |
|  | GAG GAG AAG CCC GGT CTC GAG TCA GAC TCC CTC TTC CTC C |
| pTriEx4-MAGEB1(aa63-315) | GAC GAC GAC AAG ATG CCC ATG GGA GCT CCA CCC ACC ACC |
|  | GAG GAG AAG CCC GGT CTC GAG TCA GGC TCT CTC TTC CTC ATC |
| pTriEx4-MAGEC2(aa6-373) | GAC GAC GAC AAG ATG CCC ATG GTT CCA GGC GTT CCA TTC |
|  | GAG GAG AAG CCC GGT CTC GAG TCA CTC AGA AAA GGA GAC G |
| pTriEx4-MAGED2(aa262-482) | GAC GAC GAC AAG ATG CCC ATG GCG CTC CAG TCA TCC CAA G |
|  | GAG GAG AAG CCC GGT CTC GAG TCA ATC CGC TTC CAT CGC C |
| pTriEx4-MAGED4b(aa2-741) | GAC GAC GAC AAG ATA GCT GAG GGA AGC TTC AGC GTG C |
|  | GAG GAG AAG CCC GGT TTA ACG GTG CTG GAT CCA GGA GAA G |
| pTriEx4-MAGEF1(aa2-308) | GAC GAC GAC AAG ATA TTG CAG ACA CCA GAG AGC AGG |
|  | GAG GAG AAG CCC GGT TTA CCA GAG GTG GAT GCC GGC C |
| pTriEx4-MAGEG1(aa2-304) | GAC GAC GAC AAG ATA TTG CAA AAA CCG AGG AAC CGG G |
|  | GAG GAG AAG CCC GGT TTA AGA GGA TGG AGC TGG GCC ACT AG |
| pTriEx4-MAGEG1(aa55-292) | GAC GAC GAC AAG ATG CCC ATG GGC GGC TCG CAG GGG TCG CAG |
|  | GAG GAG AAG CCC GGT CTC GAG TCA CCT GTT CTC CTC ATC TGC |
| pTriEx4-MAGEH1(aa1-219) | GAC GAC GAC AAG ATG CCC ATG GCG ATG CCT CGG GGA CGA AAG |
|  | GAG GAG AAG CCC GGT TCA GTC GAC TTA AGG GGC GGA ATA ACC |
| pTriEx4-necdin(aa1-321) | GAC GAC GAC AAG ATG CCC ATG GGG ATG TCA GAA CAA AGT AAG G |
|  | GAG GAG AAG CCC GGT CTC GAG CTA GTC CTC AGA GAC ACT GC |
